# Supplementary figures and images for: Ligand response of guanidine-IV riboswitch at single-molecule level
Source: eLife. 2024 Dec 2;13:RP94706. doi: 10.7554/eLife.94706 (PMC11611296; doi:10.7554/eLife.94706)

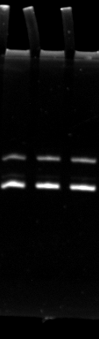

Supplement: Figure 2—figure supplement 10—source data 1. [file elife-94706-fig2-figsupp10-data1.zip › Figure 2-figure supplement 10-source data-1/Figure 2-figure supplement 10-source data-1-10C-riboG-G77C.tif]

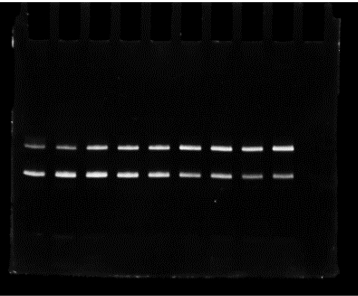

Supplement: Figure 2—figure supplement 10—source data 1. [file elife-94706-fig2-figsupp10-data1.zip › Figure 2-figure supplement 10-source data-1/Figure 2-figure supplement 10-source data-1-10C-riboG-wt.tif]

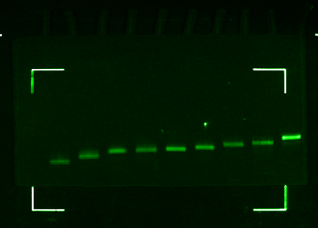

Supplement: Figure 6—figure supplement 1—source data 1. [file elife-94706-fig6-figsupp1-data1.zip › Figure 6-figure supplement 1-source data-1/Figure 6-figure supplement 1-source data 1-530 nm.tif]

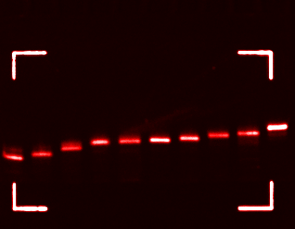

Supplement: Figure 6—figure supplement 1—source data 1. [file elife-94706-fig6-figsupp1-data1.zip › Figure 6-figure supplement 1-source data-1/Figure 6-figure supplement 1-source data 1-620 nm.tif]

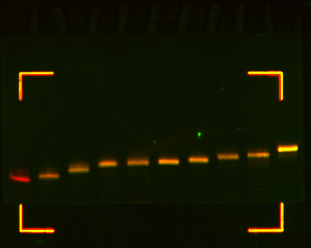

Supplement: Figure 6—figure supplement 1—source data 1. [file elife-94706-fig6-figsupp1-data1.zip › Figure 6-figure supplement 1-source data-1/Figure 6-figure supplement 1-source data 1-merge.tif]

Figure 6—figure supplement 1C

530  
nm

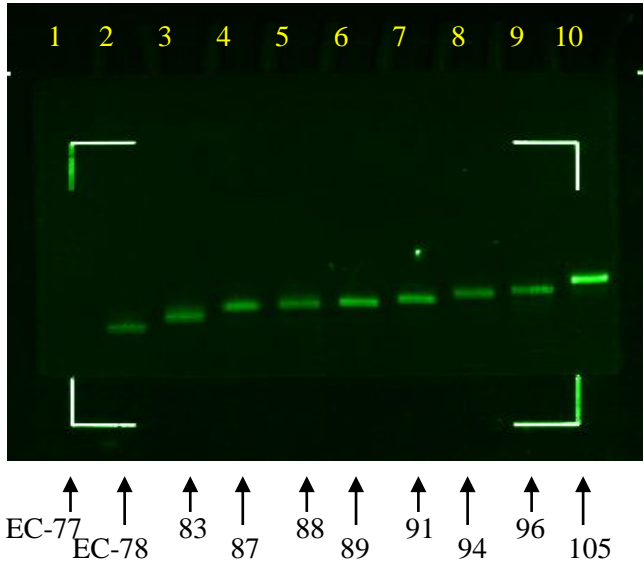

620  
nm

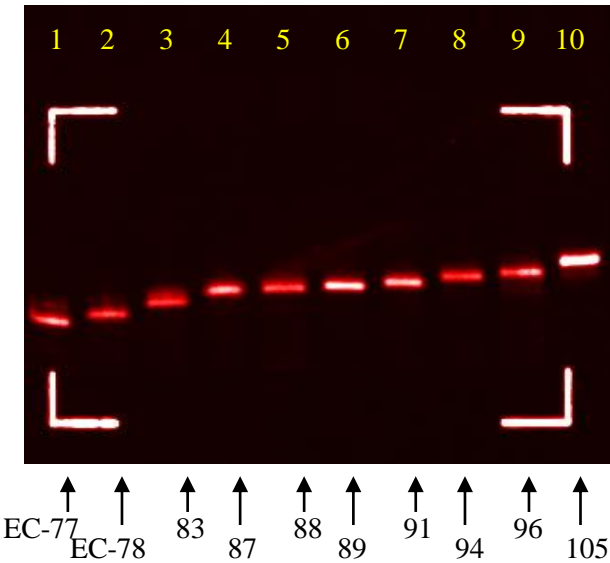

merge

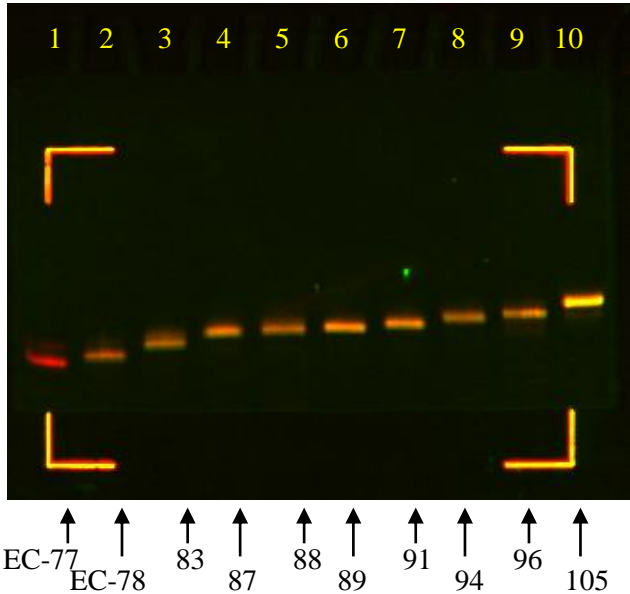

Supplement: Figure 6—figure supplement 1—source data 2. [file elife-94706-fig6-figsupp1-data2.zip › Figure 6—figure supplement 1-source data 2.pdf]

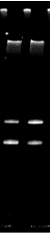

Supplement: Figure 7—source data 1. [file elife-94706-fig7-data1.zip › Figure 7-source data 1/Figure 7-source data 1- lane 1-2.tif]

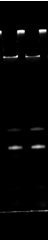

Supplement: Figure 7—source data 1. [file elife-94706-fig7-data1.zip › Figure 7-source data 1/Figure 7-source data 1- lane 15-16.tif]

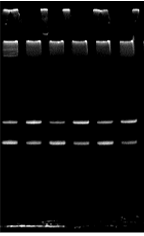

Supplement: Figure 7—source data 1. [file elife-94706-fig7-data1.zip › Figure 7-source data 1/Figure 7-source data 1- lane 3-8.tif]

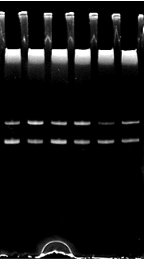

Supplement: Figure 7—source data 1. [file elife-94706-fig7-data1.zip › Figure 7-source data 1/Figure 7-source data 1- lane 9-14.tif]
