# Supplementary material for: Ligand response of guanidine-IV riboswitch at single-molecule level
Source: eLife. 2024 Dec 2;13:RP94706. doi: 10.7554/eLife.94706 (PMC11611296; doi:10.7554/eLife.94706)

Figure 2—figure supplement 10C

riboG-wt

6 mM Mg^{2+}

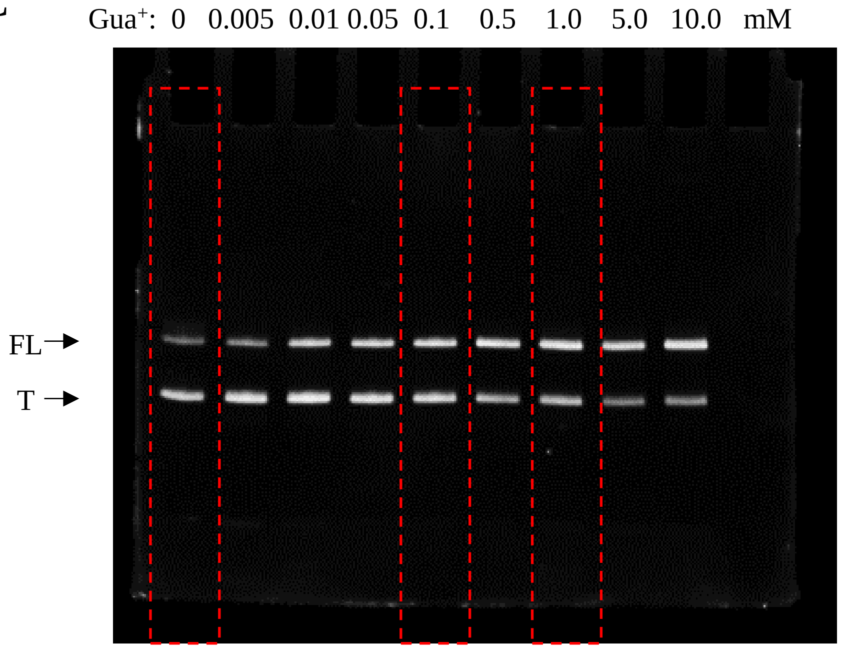

riboG-G77C

6 mM Mg^{2+}

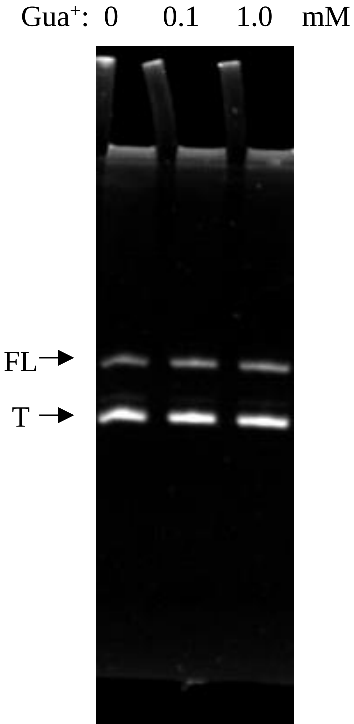

Supplement: Figure 2—figure supplement 10—source data 2. [file elife-94706-fig2-figsupp10-data2.zip › Figure 2—figure supplement 10-souce data 2.pdf]
