# Supplementary material for: Ligand response of guanidine-IV riboswitch at single-molecule level
Source: eLife. 2024 Dec 2;13:RP94706. doi: 10.7554/eLife.94706 (PMC11611296; doi:10.7554/eLife.94706)
Supplement: Figure 7—source data 2. [file elife-94706-fig7-data2.zip › Figure 7-source data 2.pdf]

Figure 7C

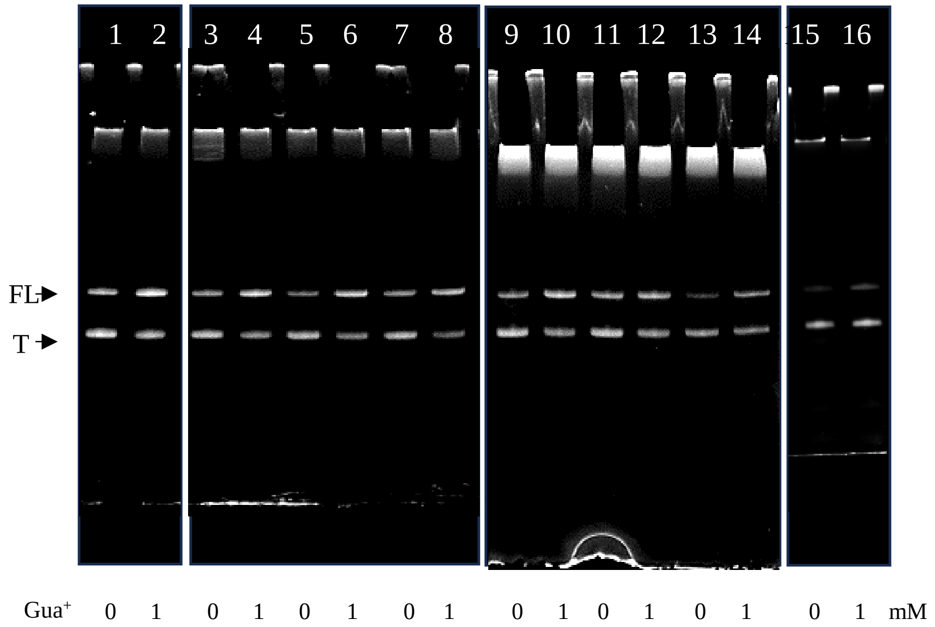

1–2: The crude products collected at the last step of 9 step PLOR in the absence and presence of 1.0 mM Gua^+.

3–4: The crude products collected at the last step of 11 step PLOR in the absence and presence of 1.0 mM Gua^+.

5–6: The crude products collected at the last step of 12 step PLOR in the absence and presence of 1.0 mM Gua^+.

7–8: The crude products collected at the last step of 13 step PLOR in the absence and presence of 1.0 mM Gua^+.

9–10: The crude products collected at the last step of 14 step PLOR in the absence and presence of 1.0 mM Gua^+.

11–12: The crude products collected at the last step of 15 step PLOR in the absence and presence of 1.0 mM Gua^+.

13–14: The crude products collected at the last step of 16 step PLOR in the absence and presence of 1.0 mM Gua^+.

15–16: The crude products collected at the step 16 and step 17 of 17 step PLOR in the absence and presence of 1.0 mM Gua^+.
