# Supplementary material for: Ligand response of guanidine-IV riboswitch at single-molecule level
Source: eLife. 2024 Dec 2;13:RP94706. doi: 10.7554/eLife.94706 (PMC11611296; doi:10.7554/eLife.94706)
Supplement: Supplementary file 1. [file elife-94706-supp1.docx]

**Table S1. The sequences of DNA templates and RNA.**

| **RNA/DNA** | **Sequence (5’-3’)** |
| --- | --- |
| riboG-apt | GGGAAGAUAUAAUCACUAAUAAGUUGCCACCGGGUAGCUUAUUUCAUGAUACUUAACUUUAUUCCUUUAGGUCUUAGUAGGAAUAAAU |
| riboG-term | GGGAAGAUAUAAUCACUAAUAAGUUGCCACCGGGUAGCUUAUUUCAUGAUACUUAACUUUAUUCCUUUAGGUCUUAGUAGGAAUAAAUGUUAGGUAUUUUUUUAU |
| full-length riboG | GGGAAGAUAUAAUCACUAAUAAGUUGCCACCGGGUAGCUUAUUUCAUGAUACUUAACUUUAUUCCUUUAGGUCUUAGUAGGAAUAAAUGUUAGGUAUUUUUUUAUGUUUAUUUUUAGGAGGAAUCACGAAAAUGAG |
| DNA template for riboG-apt | ATTTATTCCTACTAAGACCTAAAGGAATAAAGTTAAGTATCATGAAATAAGCTACCCGGTGGCAACTTATTAGTGATTATATCTTCCCTATAGTGAGTCGTATTA*TGGACTAGCTGAATCAGA* |
| DNA non-template for riboG-apt | Biotin-*TCTGATTCAGCTAGTCCA*TAATACGACTCACTATAGGGAAGATATAATCACTAATAAGTTGCCACCGGGTAGCTTATTTCATGATACTTAACTTTATTCCTTTAGGTCTTAGTAGGAATAAAT |
| DNA template for riboG-term | ATAAAAAAATACCTAACATTTATTCCTACTAAGACCTAAAGGAATAAAGTTAAGTATCATGAAATAAGCTACCCGGTGGCAACTTATTAGTGATTATATCTTCCCTATAGTGAGTCGTATTA*TGGACTAGCTGAATCAGA* |
| DNA non-template for riboG-term | Biotin-*TCTGATTCAGCTAGTCCA*TAATACGACTCACTATAGGGAAGATATAATCACTAATAAGTTGCCACCGGGTAGCTTATTTCATGATACTTAACTTTATTCCTTTAGGTCTTAGTAGGAATAAATGTTAGGTATTTTTTTAT |
| DNA template for full-length riboG | CTCATTTTCGTGATTCCTCCTAAAAATAAACATAAAAAAATACCTAACATTTATTCCTACTAAGACCTAAAGGAATAAAGTTAAGTATCATGAAATAAGCTACCCGGTGGCAACTTATTAGTGATTATATCTTCCCTATAGTGAGTCGTATTA*TGGACTAGCTGAATCAGA* |
| DNA non-template for full-length riboG | Biotin-*TCTGATTCAGCTAGTCCA*TAATACGACTCACTATAGGGAAGATATAATCACTAATAAGTTGCCACCGGGTAGCTTATTTCATGATACTTAACTTTATTCCTTTAGGTCTTAGTAGGAATAAATGTTAGGTATTTTTTTATGTTTATTTTTAGGAGGAATCACGAAAATGAG |
| Biotinylated DNA | ATTATATCTTCCC-biotin |

The T7 promoter sequences are in blue, and the DNA linker sequences to alleviate steric hindrance are in italic. The underlined sequences in RNA are hybridized with the biotinylated DNA for smFRET.

**Table S2. The sequences of riboG mutants.**

| **RNA/DNA** | **Sequence (5’-3’)** |
| --- | --- |
| riboG-G71C-apt | GGGAAGAUAUAAUCACUAAUAAGUUGCCACCGGGUAGCUUAUUUCAUGAUACUUAACUUUAUUCCUUUAGCUCUUAGUAGGAAUAAAU |
| riboG-G71C-term | GGGAAGAUAUAAUCACUAAUAAGUUGCCACCGGGUAGCUUAUUUCAUGAUACUUAACUUUAUUCCUUUAGCUCUUAGUAGGAAUAAAUGUUAGGUAUUUUUUUAU |
| full-length riboG-G71C | GGGAAGAUAUAAUCACUAAUAAGUUGCCACCGGGUAGCUUAUUUCAUGAUACUUAACUUUAUUCCUUUAGCUCUUAGUAGGAAUAAAUGUUAGGUAUUUUUUUAUGUUUAUUUUUAGGAGGAAUCACGAAAAUGAG |
| riboG-C30G-G71C-apt | GGGAAGAUAUAAUCACUAAUAAGUUGCCAGCGGGUAGCUUAUUUCAUGAUACUUAACUUUAUUCCUUUAGCUCUUAGUAGGAAUAAAU |
| riboG-C30G-G71C-term | GGGAAGAUAUAAUCACUAAUAAGUUGCCAGCGGGUAGCUUAUUUCAUGAUACUUAACUUUAUUCCUUUAGCUCUUAGUAGGAAUAAAUGUUAGGUAUUUUUUUAU |
| full-length riboG-C30G-G71C | GGGAAGAUAUAAUCACUAAUAAGUUGCCAGCGGGUAGCUUAUUUCAUGAUACUUAACUUUAUUCCUUUAGCUCUUAGUAGGAAUAAAUGUUAGGUAUUUUUUUAUGUUUAUUUUUAGGAGGAAUCACGAAAAUGAG |
| riboG-U72C-apt | GGGAAGAUAUAAUCACUAAUAAGUUGCCACCGGGUAGCUUAUUUCAUGAUACUUAACUUUAUUCCUUUAGGCCUUAGUAGGAAUAAAU |
| riboG-U72C-term | GGGAAGAUAUAAUCACUAAUAAGUUGCCACCGGGUAGCUUAUUUCAUGAUACUUAACUUUAUUCCUUUAGGCCUUAGUAGGAAUAAAUGUUAGGUAUUUUUUUAU |
| full-length riboG-U72C | GGGAAGAUAUAAUCACUAAUAAGUUGCCACCGGGUAGCUUAUUUCAUGAUACUUAACUUUAUUCCUUUAGGCCUUAGUAGGAAUAAAUGUUAGGUAUUUUUUUAUGUUUAUUUUUAGGAGGAAUCACGAAAAUGAG |
| riboG-A29G-G72C-apt | GGGAAGAUAUAAUCACUAAUAAGUUGCCGCCGGGUAGCUUAUUUCAUGAUACUUAACUUUAUUCCUUUAGGCCUUAGUAGGAAUAAAU |
| riboG-A29G-G72C-term | GGGAAGAUAUAAUCACUAAUAAGUUGCCGCCGGGUAGCUUAUUUCAUGAUACUUAACUUUAUUCCUUUAGGCCUUAGUAGGAAUAAAUGUUAGGUAUUUUUUUAU |
| full-length riboG-A29G-G72C | GGGAAGAUAUAAUCACUAAUAAGUUGCCGCCGGGUAGCUUAUUUCAUGAUACUUAACUUUAUUCCUUUAGGCCUUAGUAGGAAUAAAUGUUAGGUAUUUUUUUAUGUUUAUUUUUAGGAGGAAUCACGAAAAUGAG |
| riboG-G77C | GGGAAGAUAUAAUCACUAAUAAGUUGCCACCGGGUAGCUUAUUUCAUGAUAGUUAACUUUAUUCCUUUAGGUCUUACUAGGAAUAAAU |

The underlined sequences in RNA are hybridized with the biotinylated DNA for smFRET. The mutated nucleotides are in red.

**Table S3. Reagent usages for generating Cy3Cy5-riboG-apt.**

| **Reagent usage in a PLOR reaction (100 μL, 10 μM)** |
| --- |

| **Step 1** in the buffer (6 mM MgSO_4_, 40 mM Tris-HCl, 100 mM K_2_SO_4_, 10 mM DTT, pH 8.0) at 37 ℃ for 15 min: 10 μM DNA beads, 10 μM T7 RNAP, 960 μM ATP, 960 μM GTP, 96 μM UTP  **Steps 2-12** in the buffer (6 mM MgSO_4_, 40 mM Tris-HCl, 10 mM DTT, pH 8.0) at 25 ℃ for 10 min:  **Step 2:** 20 μM CTP, 20 μM UTP, 50 μM ATP  **Step 3:** 20 μM GTP, 20 μM UTP, 20 μM CTP  **Step 4:** 10 μM ATP, 20 μM CTP, 30 μM GTP  **Step 5:** 3 μM Cy5-UTP, 8 μM ATP, 8 μM GTP  **Step 6:** 16 μM CTP, 16 μM ATP, 48 μM UTP  **Step 7:** 8 μM GTP, 8 μM UTP, 16 μM ATP  **Step 8:** 32 μM CTP, 32 μM ATP, 80 μM UTP  **Step 9:** 16 μM GTP, 24 μM UTP, 8 μM CTP  **Step 10:** 8 μM ATP, 8 μM GTP  **Step 11:** 3 μM Cy3-UTP  **Step 12:** 16 μM GTP, 16 μM UTP, 48 μM ATP |
| --- |

**Table S4. Reagent usages for generating Cy3Cy5-riboG-term.**

| **Reagent usage in a PLOR reaction (100 μL, 10 μM)** |
| --- |

| **Step 1** in the buffer (6 mM MgSO_4_, 40 mM Tris-HCl, 100 mM K_2_SO_4_, 10 mM DTT, pH 8.0) at 37 ℃ for 15 min: 10 μM DNA beads, 10 μM T7 RNAP, 960 μM ATP, 960 μM GTP, 96 μM UTP;  **Steps 2-13** in the buffer (6 mM MgSO_4_, 40 mM Tris-HCl, 10 mM DTT, pH 8.0) at 25 ℃ for 10 min:  **Step 2:** 20 μM CTP, 20 μM UTP, 50 μM ATP;  **Step 3:** 20 μM GTP, 20 μM UTP, 20 μM CTP;  **Step 4:** 10 μM ATP, 20 μM CTP, 30 μM GTP;  **Step 5:** 3 μM Cy5-UTP, 8 μM ATP, 8 μM GTP;  **Step 6:** 16 μM CTP, 16 μM ATP, 48 μM UTP;  **Step 7:** 8 μM GTP, 8 μM UTP, 16 μM ATP;  **Step 8:** 32 μM CTP, 32 μM ATP, 80 μM UTP;  **Step 9:** 16 μM GTP, 24 μM UTP, 8 μM CTP;  **Step 10:** 8 μM ATP, 8 μM GTP;  **Step 11:** 3 μM Cy3-UTP;  **Step 12:** 16 μM GTP, 24 μM ATP;  **Step 13:** 24 μM GTP, 48 μM ATP, 104 μM UTP. |
| --- |

**Table S5. Reagent usages for generating Cy3Cy5-full-length riboG.**

| **Reagent usage in a PLOR reaction (100 μL, 10 μM)** |
| --- |

| **Step 1** in the buffer (6 mM MgSO_4_, 40 mM Tris-HCl, 100 mM K_2_SO_4_, 10 mM DTT, pH 8.0) at 37 ℃ for 15 min: 10 μM DNA beads, 10 μM T7 RNAP, 960 μM ATP, 960 μM GTP, 96 μM UTP;  **Steps 2-13** in the buffer (6 mM MgSO_4_, 40 mM Tris-HCl, 10 mM DTT, pH 8.0) at 25 ℃ for 10 min:  **Step 2:** 20 μM CTP, 20 μM UTP, 50 μM ATP;  **Step 3:** 20 μM GTP, 20 μM UTP, 20 μM CTP;  **Step 4:** 10 μM ATP, 20 μM CTP, 30 μM GTP;  **Step 5:** 3 μM Cy5-UTP, 8 μM ATP, 8 μM GTP;  **Step 6:** 16 μM CTP, 16 μM ATP, 48 μM UTP;  **Step 7:** 8 μM GTP, 8 μM UTP, 16 μM ATP;  **Step 8:** 32 μM CTP, 32 μM ATP, 80 μM UTP;  **Step 9:** 16 μM GTP, 24 μM UTP, 8 μM CTP;  **Step 10:** 8 μM ATP, 8 μM GTP;  **Step 11:** 3 μM Cy3-UTP;  **Step 12:** 16 μM GTP, 24 μM ATP;  **Step 13:** 88 μM GTP, 136 μM ATP, 16 μM CTP, 184 μM UTP. |
| --- |

**Table S6. Sequences of RNA in the ECs.**

| **ECs** | **Sequences of RNA** |
| --- | --- |
| EC-87 | GGGAAGAUAUAAUCACUAAUAAGUUGCCACCGGGUAGCUUAUUUCAUGAUACUUAACUUUAUUCCUUUAGGUCUUAGUAGGAAUAAA |
| EC-88 | GGGAAGAUAUAAUCACUAAUAAGUUGCCACCGGGUAGCUUAUUUCAUGAUACUUAACUUUAUUCCUUUAGGUCUUAGUAGGAAUAAAU |
| EC-89 | GGGAAGAUAUAAUCACUAAUAAGUUGCCACCGGGUAGCUUAUUUCAUGAUACUUAACUUUAUUCCUUUAGGUCUUAGUAGGAAUAAAUG |
| EC-91 | GGGAAGAUAUAAUCACUAAUAAGUUGCCACCGGGUAGCUUAUUUCAUGAUACUUAACUUUAUUCCUUUAGGUCUUAGUAGGAAUAAAUGUU |
| EC-94 | GGGAAGAUAUAAUCACUAAUAAGUUGCCACCGGGUAGCUUAUUUCAUGAUACUUAACUUUAUUCCUUUAGGUCUUAGUAGGAAUAAAUGUUAGG |
| EC-96 | GGGAAGAUAUAAUCACUAAUAAGUUGCCACCGGGUAGCUUAUUUCAUGAUACUUAACUUUAUUCCUUUAGGUCUUAGUAGGAAUAAAUGUUAGGUA |
| EC-105 | GGGAAGAUAUAAUCACUAAUAAGUUGCCACCGGGUAGCUUAUUUCAUGAUACUUAACUUUAUUCCUUUAGGUCUUAGUAGGAAUAAAUGUUAGGUAUUUUUUUAU |

**Table S7. Reagent usages for generating EC-87, EC-88, EC-89, EC-91, EC-94, EC-96 and EC-105.**

| **Reagent usage in a PLOR reaction (200 μL, 10 μM)** |
| --- |
| **Step 1** in the buffer (6 mM MgSO_4_, 40 mM Tris-HCl, 100 mM K_2_SO_4_, 10 mM DTT, pH 8.0) at 37 ℃ for 15 min: 10 μM DNA beads, 10 μM T7 RNAP, 960 μM ATP, 960 μM GTP, 96 μM UTP;  **Steps 2-21** in the buffer (6 mM MgSO_4_, 40 mM Tris-HCl, 10 mM DTT, pH 8.0) at 25 ℃ for 10 min:  **Step 2:** 20 μM CTP, 20 μM UTP, 50 μM ATP;  **Step 3:** 20 μM GTP, 20 μM UTP, 20 μM CTP;  **Step 4:** 10 μM ATP, 20 μM CTP, 30 μM GTP;  **Step 5:** 3 μM Cy5-UTP, 8 μM ATP, 8 μM GTP;  **Step 6:** 16 μM CTP, 16 μM ATP, 48 μM UTP;  **Step 7:** 8 μM GTP, 8 μM UTP, 16 μM ATP;  **Step 8:** 32 μM CTP, 32 μM ATP, 80 μM UTP;  **Step 9:** 16 μM GTP, 24 μM UTP, 8 μM CTP;  **Step 10:** 8 μM ATP, 8 μM GTP;  **Step 11:** 3 μM Cy3-UTP;  **Step 12:** 16 μM GTP, 24 μM ATP;  **Step 13:** 8 μM UTP;  **Step 14:** 24 μM ATP (the dissociated EC-87 was used for smFRET);  **Step 15:** 8 μM UTP (the dissociated EC-88 was used for smFRET);  **Step 16:** 8 μM GTP (the dissociated EC-89 was used for smFRET);  **Step 17:** 16 μM UTP (the dissociated EC-91 was used for smFRET);  **Step 18:** 8 μM ATP, 16 μM GTP (the dissociated EC-94 was used for smFRET);  **Step 19:** 8 μM UTP;  **Step 20:** 8 μM ATP (the dissociated EC-96 was used for smFRET);  **Step 21:** 64 μM UTP, 8 μM ATP (the dissociated EC-105 was used for smFRET). |

**Table S8. Reagent usages for termination assays by 8 step-PLOR.**

| **Reagent usage in PLOR reactions (100 μL, 3 μM)** |
| --- |
| **Step 1** in the buffer (6 mM MgSO_4_, 40 mM Tris-HCl, 100 mM K_2_SO_4_, 10 mM DTT, pH 8.0) at 37 ℃ for 15 min: 3 μM DNA beads, 3 μM T7 RNAP, 288 μM ATP, 288 μM GTP, 28.8 μM UTP;  **Steps 2-8** in the buffer (40 mM Tris-HCl, 10 mM DTT, pH 8.0) at 25 ℃ for 10 min:  **Step 2:** 6 μM CTP, 6 μM UTP, 15 μM ATP, 6 mM MgSO_4_;  **Step 3:** 6 μM GTP, 6 μM UTP, 6 μM CTP, 6 mM MgSO_4_;  **Step 4:** 3 μM ATP, 6 μM CTP, 9 μM GTP, 6 mM MgSO_4_;  **Step 5:** 0.9 μM UTP, 2.4μM ATP, 2.4μM GTP, 6 mM MgSO_4_;  **Step 6:** 4.8 μM CTP, 4.8 μM ATP, 14.4 μM UTP, 6 mM MgSO_4_;  **Step 7:** 2.4 μM GTP, 2.4 μM UTP, 4.8 μM ATP, 6 mM MgSO_4_;  **Step 8:** 6.3 μM CTP, 22.5 μM ATP, 33.3 μM UTP, 14.4 μM GTP, 6 mM MgSO_4_, 0­­­–10 mM Gua^+^; |

**Table S9. Reagent usages for termination assay by 9 step-PLOR.**

| **Reagent usage in PLOR reactions (100 μL, 3 μM)** |
| --- |

| **Step 1** in the buffer (6 mM MgSO_4_, 40 mM Tris-HCl, 100 mM K_2_SO_4_, 10 mM DTT, pH 8.0) at 37 ℃ for 15 min: 3μM DNA beads, 3 μM T7 RNAP, 288 μM ATP, 288 μM GTP, 28.8 μM UTP;  **Steps 2-9** in the buffer (6 mM MgSO_4_, 40 mM Tris-HCl, 10 mM DTT, pH 8.0) at 25 ℃ for 10 min:  **Step 2:** 6 μM CTP, 6 μM UTP, 15 μM ATP;  **Step 3:** 6 μM GTP, 6 μM UTP, 6 μM CTP;  **Step 4:** 3 μM ATP, 6 μM CTP, 9 μM GTP;  **Step 5:** 0.9 μM UTP, 2.4μM ATP, 2.4μM GTP;  **Step 6:** 4.8 μM CTP, 4.8 μM ATP, 14.4 μM UTP;  **Step 7:** 2.4 μM GTP, 2.4 μM UTP, 4.8 μM ATP;  **Step 8:** 9.6 μM CTP, 24 μM UTP, 9.6 μM ATP;  **Step 9:** 2.7 μM CTP, 18.9 μM ATP, 24.3 μM UTP, 14.4 μM GTP, 0­­­ or 1 mM Gua^+^. |
| --- |

**Table S10. Reagent usages for termination assay by 11 step-PLOR.**

| **Reagent usage in PLOR reactions (100 μL, 3 μM)** |
| --- |

| **Step 1** in the buffer (6 mM MgSO_4_, 40 mM Tris-HCl, 100 mM K_2_SO_4_, 10 mM DTT, pH 8.0) at 37 ℃ for 15 min: 3 μM DNA beads, 3 μM T7 RNAP, 288 μM ATP, 288 μM GTP, 28.8 μM UTP;  **Steps 2-11** in the buffer (6 mM MgSO_4_, 40 mM Tris-HCl, 10 mM DTT, pH 8.0) at 25 ℃ for 10 min:  **Step 2:** 6 μM CTP, 6 μM UTP, 15 μM ATP;  **Step 3:** 6 μM GTP, 6 μM UTP, 6 μM CTP;  **Step 4:** 3 μM ATP, 6 μM CTP, 9 μM GTP;  **Step 5:** 0.9 μM UTP, 2.4μM ATP, 2.4μM GTP;  **Step 6:** 4.8 μM CTP, 4.8 μM ATP, 14.4 μM UTP;  **Step 7:** 2.4 μM GTP, 2.4 μM UTP, 4.8 μM ATP;  **Step 8:** 9.6 μM CTP, 24 μM UTP, 9.6 μM ATP;  **Step 9:** 4.8 μM GTP, 7.2 μM UTP, 2.4 μM CTP;  **Step 10:** 2.4 μM GTP, 2.4 μM ATP;  **Step 11:** 1.8 μM CTP, 18 μM ATP, 21.6 μM UTP, 11.7 μM GTP, 0­­­ or 1 mM Gua^+^. |
| --- |

**Table S11. Reagent usages for termination assay by 12 step-PLOR.**

| **Reagent usage in PLOR reactions (100 μL, 3 μM)** |
| --- |
| **Step 1** in the buffer (6 mM MgSO_4_, 40 mM Tris-HCl, 100 mM K_2_SO_4_, 10 mM DTT, pH 8.0) at 37 ℃ for 15 min: 3 μM DNA beads, 3 μM T7 RNAP, 288 μM ATP, 288 μM GTP, 28.8 μM UTP;  **Steps 2-12** in the buffer (6 mM MgSO_4_, 40 mM Tris-HCl, 10 mM DTT, pH 8.0) at 25 ℃ for 10 min:  **Step 2:** 6 μM CTP, 6 μM UTP, 15 μM ATP;  **Step 3:** 6 μM GTP, 6 μM UTP, 6 μM CTP;  **Step 4:** 3 μM ATP, 6 μM CTP, 9 μM GTP;  **Step 5:** 0.9 μM UTP, 2.4μM ATP, 2.4μM GTP;  **Step 6:** 4.8 μM CTP, 4.8 μM ATP, 14.4 μM UTP;  **Step 7:** 2.4 μM GTP, 2.4 μM UTP, 4.8 μM ATP;  **Step 8:** 9.6 μM CTP, 24 μM UTP, 9.6 μM ATP;  **Step 9:** 4.8 μM GTP, 7.2 μM UTP, 2.4 μM CTP;  **Step 10:** 2.4 μM GTP, 2.4 μM ATP;  **Step 11:** 2.4 μM UTP;  **Step 12:** 1.8 μM CTP, 18 μM ATP, 20.7 μM UTP, 11.7 μM GTP, 0­­­ or 1 mM Gua^+^. |

**Table S12. Reagent usages for termination assay by 13 step-PLOR.**

| **Reagent usage in PLOR reactions (100 μL, 3 μM)** |
| --- |
| **Step 1** in the buffer (6 mM MgSO_4_, 40 mM Tris-HCl, 100 mM K_2_SO_4_, 10 mM DTT, pH 8.0) at 37 ℃ for 15 min: 3 μM DNA beads, 3 μM T7 RNAP, 288 μM ATP, 288 μM GTP, 28.8 μM UTP;  **Steps 2-13** in the buffer (6 mM MgSO_4_, 40 mM Tris-HCl, 10 mM DTT, pH 8.0) at 25 ℃ for 10 min:  **Step 2:** 6 μM CTP, 6 μM UTP, 15 μM ATP;  **Step 3:** 6 μM GTP, 6 μM UTP, 6 μM CTP;  **Step 4:** 3 μM ATP, 6 μM CTP, 9 μM GTP;  **Step 5:** 0.9 μM UTP, 2.4μM ATP, 2.4μM GTP;  **Step 6:** 4.8 μM CTP, 4.8 μM ATP, 14.4 μM UTP;  **Step 7:** 2.4 μM GTP, 2.4 μM UTP, 4.8 μM ATP;  **Step 8:** 9.6 μM CTP, 24 μM UTP, 9.6 μM ATP;  **Step 9:** 4.8 μM GTP, 7.2 μM UTP, 2.4 μM CTP;  **Step 10:** 2.4 μM GTP, 2.4 μM ATP;  **Step 11:** 2.4 μM UTP;  **Step 12:** 4.8 μM GTP, 7.2 μM ATP;  **Step 13:** 1.8 μM CTP, 15.3 μM ATP, 20.7 μM UTP, 9.9 μM GTP, 0­­­ or 1 mM Gua^+^. |

**Table S13. Reagent usages for termination assay by 14 step-PLOR.**

| **Reagent usage in PLOR reactions (100 μL, 3 μM)** |
| --- |
| **Step 1** in the buffer (6 mM MgSO_4_, 40 mM Tris-HCl, 100 mM K_2_SO_4_, 10 mM DTT, pH 8.0) at 37 ℃ for 15 min: 3 μM DNA beads, 3 μM T7 RNAP, 288 μM ATP, 288 μM GTP, 28.8 μM UTP;  **Steps 2-14** in the buffer (6 mM MgSO_4_, 40 mM Tris-HCl, 10 mM DTT, pH 8.0) at 25 ℃ for 10 min:  **Step 2:** 6 μM CTP, 6 μM UTP, 15 μM ATP;  **Step 3:** 6 μM GTP, 6 μM UTP, 6 μM CTP;  **Step 4:** 3 μM ATP, 6 μM CTP, 9 μM GTP;  **Step 5:** 0.9 μM UTP, 2.4μM ATP, 2.4μM GTP;  **Step 6:** 4.8 μM CTP, 4.8 μM ATP, 14.4 μM UTP;  **Step 7:** 2.4 μM GTP, 2.4 μM UTP, 4.8 μM ATP;  **Step 8:** 9.6 μM CTP, 24 μM UTP, 9.6 μM ATP;  **Step 9:** 4.8 μM GTP, 7.2 μM UTP, 2.4 μM CTP;  **Step 10:** 2.4 μM GTP, 2.4 μM ATP;  **Step 11:** 2.4 μM UTP;  **Step 12:** 4.8 μM GTP, 7.2 μM ATP;  **Step 13:** 4.8 μM UTP, 7.2 μM ATP;  **Step 14:** 1.8 μM CTP, 12.6 μM ATP, 18.9 μM UTP, 9.9 μM GTP, 0­­­ or 1 mM Gua^+^. |

**Table S14. Reagent usages for termination assay by 15 step-PLOR.**

| **Reagent usage in PLOR reactions (100 μL, 3 μM)** |
| --- |
| **Step 1** in the buffer (6 mM MgSO_4_, 40 mM Tris-HCl, 100 mM K_2_SO_4_, 10 mM DTT, pH 8.0) at 37 ℃ for 15 min: 3 μM DNA beads, 3 μM T7 RNAP, 288 μM ATP, 288 μM GTP, 28.8 μM UTP;  **Steps 2-15** in the buffer (6 mM MgSO_4_, 40 mM Tris-HCl, 10 mM DTT, pH 8.0) at 25 ℃ for 10 min:  **Step 2:** 6 μM CTP, 6 μM UTP, 15 μM ATP;  **Step 3:** 6 μM GTP, 6 μM UTP, 6 μM CTP;  **Step 4:** 3 μM ATP, 6 μM CTP, 9 μM GTP;  **Step 5:** 0.9 μM UTP, 2.4μM ATP, 2.4μM GTP;  **Step 6:** 4.8 μM CTP, 4.8 μM ATP, 14.4 μM UTP;  **Step 7:** 2.4 μM GTP, 2.4 μM UTP, 4.8 μM ATP;  **Step 8:** 9.6 μM CTP, 24 μM UTP, 9.6 μM ATP;  **Step 9:** 4.8 μM GTP, 7.2 μM UTP, 2.4 μM CTP;  **Step 10:** 2.4 μM GTP, 2.4 μM ATP;  **Step 11:** 2.4 μM UTP;  **Step 12:** 4.8 μM GTP, 7.2 μM ATP;  **Step 13:** 4.8 μM UTP, 7.2 μM ATP;  **Step 14:** 2.4 μM GTP, 4.8 μM UTP;  **Step 15:** 1.8 μM CTP, 12.6 μM ATP, 17.1 μM UTP, 9.0 μM GTP, 0­­­ or 1 mM Gua^+^. |

**Table S15. Reagent usages for termination assay by 16 step-PLOR.**

| **Reagent usage in PLOR reactions (100 μL, 3 μM)** |
| --- |
| **Step 1** in the buffer (6 mM MgSO_4_, 40 mM Tris-HCl, 100 mM K_2_SO_4_, 10 mM DTT, pH 8.0) at 37 ℃ for 15 min: 3 μM DNA beads, 3 μM T7 RNAP, 288 μM ATP, 288 μM GTP, 28.8 μM UTP;  **Steps 2-16** in the buffer (6 mM MgSO_4_, 40 mM Tris-HCl, 10 mM DTT, pH 8.0) at 25 ℃ for 10 min:  **Step 2:** 6 μM CTP, 6 μM UTP, 15 μM ATP;  **Step 3:** 6 μM GTP, 6 μM UTP, 6 μM CTP;  **Step 4:** 3 μM ATP, 6 μM CTP, 9 μM GTP;  **Step 5:** 0.9 μM UTP, 2.4μM ATP, 2.4μM GTP;  **Step 6:** 4.8 μM CTP, 4.8 μM ATP, 14.4 μM UTP;  **Step 7:** 2.4 μM GTP, 2.4 μM UTP, 4.8 μM ATP;  **Step 8:** 9.6 μM CTP, 24 μM UTP, 9.6 μM ATP;  **Step 9:** 4.8 μM GTP, 7.2 μM UTP, 2.4 μM CTP;  **Step 10:** 2.4 μM GTP, 2.4 μM ATP;  **Step 11:** 2.4 μM UTP;  **Step 12:** 4.8 μM GTP, 7.2 μM ATP;  **Step 13:** 4.8 μM UTP, 7.2 μM ATP;  **Step 14:** 2.4 μM GTP, 4.8 μM UTP;  **Step 15:** 4.8 μM GTP, 2.4 μM ATP;  **Step 16:** 1.8 μM CTP, 11.7 μM ATP, 17.1 μM UTP, 7.2 μM GTP, 0­­­ or 1 mM Gua^+^. |

**Table S16. Reagent usages for termination assay by 17 step-PLOR.**

| **Reagent usage in PLOR reactions (100 μL, 3 μM)** |
| --- |
| **Step 1** in the buffer (6 mM MgSO_4_, 40 mM Tris-HCl, 100 mM K_2_SO_4_, 10 mM DTT, pH 8.0) at 37 ℃ for 15 min: 3 μM DNA beads, 3 μM T7 RNAP, 288 μM ATP, 288 μM GTP, 28.8 μM UTP;  **Steps 2-17** in the buffer (6 mM MgSO_4_, 40 mM Tris-HCl, 10 mM DTT, pH 8.0) at 25 ℃ for 10 min:  **Step 2:** 6 μM CTP, 6 μM UTP, 15 μM ATP;  **Step 3:** 6 μM GTP, 6 μM UTP, 6 μM CTP;  **Step 4:** 3 μM ATP, 6 μM CTP, 9 μM GTP;  **Step 5:** 0.9 μM UTP, 2.4μM ATP, 2.4μM GTP;  **Step 6:** 4.8 μM CTP, 4.8 μM ATP, 14.4 μM UTP;  **Step 7:** 2.4 μM GTP, 2.4 μM UTP, 4.8 μM ATP;  **Step 8:** 9.6 μM CTP, 24 μM UTP, 9.6 μM ATP;  **Step 9:** 4.8 μM GTP, 7.2 μM UTP, 2.4 μM CTP;  **Step 10:** 2.4 μM GTP, 2.4 μM ATP;  **Step 11:** 2.4 μM UTP;  **Step 12:** 4.8 μM GTP, 7.2 μM ATP;  **Step 13:** 4.8 μM UTP, 7.2 μM ATP;  **Step 14:** 2.4 μM GTP, 4.8 μM UTP;  **Step 15:** 4.8 μM GTP, 2.4 μM ATP;  **Step 16:** 21.6 μM UTP, 4.8 μM ATP;  **Step 17:** 1.8 μM CTP, 9.9 μM ATP, 9.0 μM UTP, 7.2 μM GTP, 0­­­ or 1 mM Gua^+^. |
